# Prevalence of Muscular Skeletal Disorders among Qualified Dental Assistants

**DOI:** 10.3390/ijerph17103490

**Published:** 2020-05-16

**Authors:** Daniela Ohlendorf, Yvonne Haas, Antonia Naser, Jasmin Haenel, Laura Maltry, Fabian Holzgreve, Christina Erbe, Werner Betz, Eileen M. Wanke, Dörthe Brüggmann, Albert Nienhaus, David A. Groneberg

**Affiliations:** 1 Institute of Occupational Medicine, Social Medicine and Environmental Medicine, Goethe-University, Theodor-Stern-Kai 7, 60596 Frankfurt am Main, Germany; yvonne_haas@gmx.de (Y.H.); antonia_naser@web.de (A.N.); j.lampe@med.uni-frankfurt.de (J.H.); maltry@med.uni-frankfurt.de (L.M.); holzgreve@med.uni-frankfurt.de (F.H.); wanke@med.uni-frankfurt.de (E.M.W.); brueggmann@med.uni-frankfurt.de (D.B.); groneberg@med.uni-frankfurt.de (D.A.G.); 2 Department of Orthodontics, School of Dentistry, University Medical Centre of the Johannes Gutenberg University Mainz, 55131 Mainz, Germany; erbe@uni-mainz.de; 3 Institute of Dentistry, Goethe-University, Theodor-Stern-Kai 7, 60590 Frankfurt am Main, Germany; w.betz@em.uni-frankfurt.de; 4 Competence Center for Epidemiology and Health Services Research for Healthcare Professionals (CVcare), University Medical Center Hamburg-Eppendorf (UKE), Principles of Prevention and Rehabilitation Department (GPR), Institute for Statutory Accident Insurance and Prevention in the Health and Welfare Services (BGW), 20251 Hamburg, Germany; albert.nienhaus@bgw-online.de

**Keywords:** MSD, musculoskeletal, pain, prevalence, dental assistants, dental profession, dentist, dental education, questionnaire

## Abstract

The occupation of dental assistants (DAs) involves many health risks of the musculoskeletal system due to static and prolonged work, which can lead to musculoskeletal disorders (MSDs). The aim of the study was to investigate the prevalence of MSDs in DAs in Germany. Methods: For this purpose, an online questionnaire analyzed 406 (401 female participants and 5 male participants, 401w/5m) DAs. It was based on the Nordic Questionnaire (lifetime, 12-month, and seven-day MSDs’ prevalence separated into neck, shoulder, elbow, wrist, upper back, lower back, hip, knee, and ankle), and occupational and sociodemographic questions as well as questions about specific medical conditions. Results: 98.5% of the participants reported complaints of at least one body region in their lives, 97.5% reported at least one complaint in the last 12 months and 86.9% affirmed at least one complaint in the last seven days. For lifetime, 12-month and seven-day prevalence, the neck was the region that was most affected followed by the shoulder, the upper back and the lower back. Conclusion: The prevalence of MSDs among German (female) DAs was very high. The most affected area is the neck, followed by the shoulder, the lower back, and the upper back. It, therefore, seems necessary to devote more attention to ergonomics at the working practice of DAs as well in education and in dental work.

## 1. Introduction

The physical demand on dental healthcare professionals is high since their working area—the patient’s mouth—is small and narrow and treating patients requires long static seated postures and repetitive work of the arms [1,2]. Lietz et al. [3] identified numerous risk factors for the physical well-being of dental healthcare professionals in Western countries like vibrating instruments, working postures with a high physical demand, a large number of patients to treat, and extensive administrative work. Also, a study compared 974 occupations regarding their intrinsic health risks and identified the daily work of dental healthcare professionals as the most impactful on physical health [4]. Exposures to contamination, disease, and infection, as well as the extended time spent sitting, were the main reasons for the classification as an “unhealthy profession” [4]. Musculoskeletal disorders (MSDs) such as tendinitis or tension neck syndrome result from extended periods of sitting in forced body postures with static torsions, flexions, and lateral flexions of the torso [5,6,7,8]. MSDs do not only lead to limitations in everyday life, but also create a high disease burden in a work-related context [9]. They might lead to sickness, absenteeism, incapacity for work, or early retirement [10,11]. In 2018, “conditions of the musculoskeletal system”—specifically diseases of the back—were the most frequent diagnoses for an incapacity to work in Germany. MSDs were linked to 20.9% of all days employees were absent from the workplace [12,13]. 

Various international studies assessed MSDs among dental staff by questionnaires and showed generally a high prevalence worldwide [8,9,14,15,16,17,18,19,20,21,22,23,24,25]. In Saudi Arabia, 85.0% of the dentists (D), dental assistants (DAs), dental hygienists (DH), and dental technicians (DT) noticed MSDs after starting their occupation [20]. In Canada, Ds, DHs, and DAs were asked about pain in the last 12 months [16]. Female DAs were most commonly affected. In this group, the MDS prevalence in the lower back region was 40.0%, 36% in the shoulder region, 35% in the neck region, and 33.0% in the upper back [16]. Data collected from a cohort of German *dentists* reported that 86.7% had suffered from spinal problems the previous year, particularly in the neck and upper back [14]. Further, a one-year prevalence of MSDs was surveyed among Danish dentists. The authors reported disease of the shoulders in 88%, the lower back in 63.0%, and the neck in 50.0% (modified Nordic Questionnaire) [22]. The 12-month prevalence of MSDs among male dentists in Israel was slightly lower with 55.0% for the back and 38.3% for the neck (modified Nordic Questionnaire) [8]. Chinese dentists complained of symptoms in the neck (83.8%) and the shoulders (40.1%), but also the hand (18.4%) and the elbow (15.1%) (as surveyed by the modified Nordic Questionnaire) [9].

In India, 56.2% of dentists reported sensitivity to pain in the upper trapezius region and 42.6% in the cervical region [23]. Among Czech dentists, 96.6% of respondents to the modified Nordic Questionnaire experienced at least one MSD in the past year; 66.3% reported medium to high pain intensities [18]. The most common areas for pain among Nepalese Ds were the back (80.0%), followed by the neck (58.8%) and the shoulder (47.0%) [26]. In Spain, 79.8% of respondents (faculty members and post-graduate students) reported MSDs in the last six months [17]. The neck region was most frequently affected in 58.0% of the respondents followed by the lumbar region (52.7%), the back (40.5%), the wrist (27.1%), and the shoulder (24.3%) [17]. Among Australian dental students, the MSDs prevalence in the neck was 64.3%, in the lower back 57.9% and in the shoulder 48.4% [24]. 

A study in Thailand investigated the prevalences of MSDs specifically in dental assistants. Of them, 60.8% of the DAs confirmed pain in the shoulder region, 60.8% in the neck region, and 54.1% in the lower back region. Also, the lower extremities were affected. Here, 62% reported pain in the legs, 48.0% in the feet, and 43% in the knees [21]. When comparing the different dental professions, Morse et al. [25] reported similar rates of MSDs in Ds, DHs, and DAs in the USA. Among Ds, prevalences of MSDs in the last 12 months were found to be 26.0–73.0% in the neck and 20.0–65.0% in the shoulders; among DHs, 54–83% in the neck and 35–76% in the shoulders; and Das, 38.0–62.0% in the neck and 27.0–62.0% in the shoulders. The same result was found for female Ds, DHs, and DAs in Canada. For example, female Ds showed similar MSDs prevalences in the neck (36.0%), in the upper back (30.0%), in the lower back (23.0%), and in the shoulder (35.0%) [16]. Also, a survey of the prevalence of upper extremity disorders among female dental healthcare professionals in Sweden found no significant difference [19]. 

Dental healthcare professionals work in an unhealthy environment [4]. Musculoskeletal disorders are frequently experienced among all professional groups in the dental field. Prevalences of the specific complaints are generally high among male and female Ds, DHs, and DAs. In this occupational group, MSDs create a huge burden for the individual and public health. Some evidence showed that specifically the DAs are prone to experience work-related symptoms. For example, female dental assistants in Canada had the highest prevalence of neck pain among all other dental professions [16]. Although the body of literature examining dentists is large, data on MSD prevalences of dental assistants are scarce. Especially for Germany, no evidence exists so far. Therefore, the aim of the present study was to collect data on current prevalences (in relation to lifetime, the last 12 months, and the last seven days) of MSDs in DAs at work and in apprenticeship in Germany using a modified Nordic Questionnaire.

## 2. Materials and Methods

We designed this observational study as a cross-sectional study. Data were collected between May 2018 and May 2019 using an anonymous online questionnaire, which was distributed via the SoSci Survey platform [27]. 

### 2.1. Subjects

For this study, we identified a study population of 2548 individuals. They were approached by different recruitment strategies. The subjects received a study invitation by email containing a link to the questionnaire. Of them, 520 participants finished the questionnaire, and a sample of 406 was included in the analysis according to the inclusion and exclusion criteria. Inclusion criteria for the study were the following: A minimum age of 18 years, a job or apprentice position in Germany, and the membership of a professional DA society or an apprentice DA contract. Respondents who were not classified as DAs per official definition of the society (e.g., dental hygienists) were excluded. Furthermore, employees in administration, industry, and the dental laboratory were excluded. Uncompleted questionnaires were also not considered for the analysis. 

Of the 406 participants, 401 identified as female and five as male DAs. Of these, 79.0% were already in employment and 21.0% were still in apprenticeship. The participants were aged between 18 and 68 years. The average age was 31.1 years with a standard deviation of 10.2 years. All participants worked in the following areas according to their employers’ specialization: Generalist (58.6%), oral surgery/maxillofacial surgery (10.1%), endodontology (5.4%), orthodontics (8.6%), prosthetics (0.7%), pediatric dentistry (0.7%), periodontology (1.2%), DAs who mentioned dental hygiene as their specialization (10.6%), and others (2.9%).

A declaration of informed consent had to be accepted online at the beginning of the survey. The study was approved by the local medical ethics committee of the medical faculty (Goethe University Frankfurt, No. 356/17).

### 2.2. Questionnaire 

The questionnaire of this survey was a modified version of the validated Nordic Questionnaire [28,29]. We collected variables on socio-demographic characteristics (gender, age, education in Germany, size, weight, and handedness) as well as information on the working environment and the type of employment (professional (employed or in apprenticeship) group, area of the employers’ specialization, professional experience in years, average total working time by the week in hours, average treatment time by the week in hours, average administrative time by the week in hours, job change, federate state of dental office, practice type). This pool of questions was based on the Meyer questionnaire [14]. According to the Nordic Questionnaire [28,29], we requested typical symptoms associated with MSDs as our primary outcome. These complaints were inquired according to the affected body areas and the duration of the symptoms such lifetime, 12-month, and seven-day MSDs. In addition, we queried specific medical conditions, which occur frequently in the dental profession.

The Nordic Questionnaire, developed by Kuorinka et al. [29], investigates musculoskeletal complaints.

Internationally, this validated questionnaire with 28 multiple-choice questions is used for studies on various occupational groups, such as administrative staff [30,31,32,33], factory workers [34,35,36,37], or medical service providers [38,39,40,41]. The questions investigate the existence of MSDs during the last seven days and the last 12 months. The symptoms are queried separately for each of the nine body regions (neck, shoulder, elbow, wrist, thoracic spine, lumbar spine, hip, knee, and ankle). In addition, the questionnaire examines the regions of the neck, the shoulder, and the lower back in more detail and asks thereby for the lifetime prevalence of MSDs, professional and private consequences of MSDs, the duration of the problems, possible accidents, and therapies of each of the three regions. In this study, only the questions about the prevalence were asked. The questionnaire by Meyer et al. [14] examined the workload of dentists in private practice in Germany. Sociodemographic, the questions on workplace and occupation, were modified for the present study. 

Furthermore, specific medical conditions, which can be associated with the physical demands of an occupation in dental healthcare, were queried [1,16,42,43,44,45,46,47,48,49]. The data include questions about chronic inflammatory joint diseases such as rheumatism and osteoarthritis; carpal and cubital tunnel syndromes; cervical, thoracic, and lumbar spine syndromes; tendovaginitis; flexor tendovaginitis; and disc prolapse. 

We validated our questionnaire using feedback from 13 volunteer participants within the target population. The pretest included in the end additional questions that asked for suggestions of improvement and general remarks. After the session, we made amendments to our questionnaire to ensure understanding and practicability of the questions. These contained improvements of the layout and the formatting, removing questions, adding of additional possible answers, and adjustments of questions for a better understanding.

### 2.3. Recruitment

We recruited the study participants via different access routes. On the one hand, the dental chambers nationwide were made aware of the study with information and flyers. The project was then shared with dental staff via internal networks. On the other hand, the study was distributed via various Germany-wide social networks of Ds, DAs, and DAs in apprenticeship. Furthermore, flyers were sent to apprenticeship schools of DAs in the federate state “Hessen” (Germany), and flyers were distributed at the German Dentists’ Day in Frankfurt am Main (Germany) 9 October 2018–11 October 2018 and at the “International Dental Show (IDS)” in Cologne (Germany) on 15 March 2019 and 16 March 2019. These two events were considered the largest events for dentists and DAs in Germany. The study information was also made public via articles in the specialist journal “Die Zahnarzt Woche (DZW)” and the journal “Zahnärztliche Mitteilungen (ZM)”. The problem with acquiring DAs is that they do not have a superordinate lobby. All dental assistants (regardless of the educational situation) receive relevant information from their boss, the dentist. Therefore, an acquisition is more or less only possible indirectly. To address DAs directly, we selected the two largest German trade fairs.

### 2.4. Data Editing and Analysis

The data were exported to “Microsoft Excel 16.5” [50] via the SoSci Survey platform [27]. The subsequent data processing and encoding of open answers was done via Excel. The data were checked and processed with regard to the logic in the response behavior. In the case of inadequate classification in the occupational group or confusion of the occupational group with the area of the employers’ specialization, data were sorted correctly. Specializations were also grouped together under the designation “Other” and, in case of multiple entries, the new groups, prosthetics, pediatric dentistry, and periodontology, as well as dental hygiene, were formed. With the designation “dental hygiene”, only those DAs were selected who indicated this as their specialty. DAs who indicated dental hygienist as their profession were excluded. The answer to the question about the total working time in hours per week, which was not consistent with the sum of the questions about treatment and administration time in hours per week, was left out of the evaluation. In addition, obvious errors in body height were corrected, insofar as the number did not match the measurement unit. But in case of any uncertainty the answers were not used in the analysis. If only the seven-day prevalence or the 12-month prevalence of one of the body regions was given, the lifetime prevalence was subsequently selected accordingly. In the case of complaints in regions indicated as “right” and “left” but not “both”, “both” was selected retrospectively. If, in addition, “no complaints” was indicated and a complaint was also indicated, the selection "no complaints" was removed. 

The data were evaluated descriptively with “IBM Statistics SPSS 26” [51] via dispersion measures and frequencies. Metric data were checked for normal distribution using the Kolmogorov–Smirnov test. For all non-normally distributed metric data, the median and interquartile distances were computed. In addition, a Spearman’s rank correlation coefficient was calculated between age and work experience, age and the number of reported MSDs, and age and the presence of specific conditions. Furthermore, correlations between Body-Mass-Index (BMI) as well as the height with the number of reported MSDs and selected diseases were examined.

When calculating the correlations with specific medical conditions and lifetime prevalence, the partial rank correlation over the interference factor age was calculated, since there were no comparable periods of time. 

## 3. Results

### 3.1. Study Population—Demographic Data

Table 1 shows the sociodemographic data of the total n = 406 (100%) DAs and DAs in apprenticeship, whereby 98.9% of the participants were female. The response rate was 20.4%. 

### 3.2. Study Population—Work-Related Questions

Table 2 describes the workplace-related parameters of the DAs. Of the respondents, 79.3% were employed and 20.7% were still in apprenticeship and, therefore, had not yet completed vocational training. The median of professional experience was 8 years. The median treatment time was 30 hours per week with a total working time of 38.5 hours per week on average.

**Figure 1:**
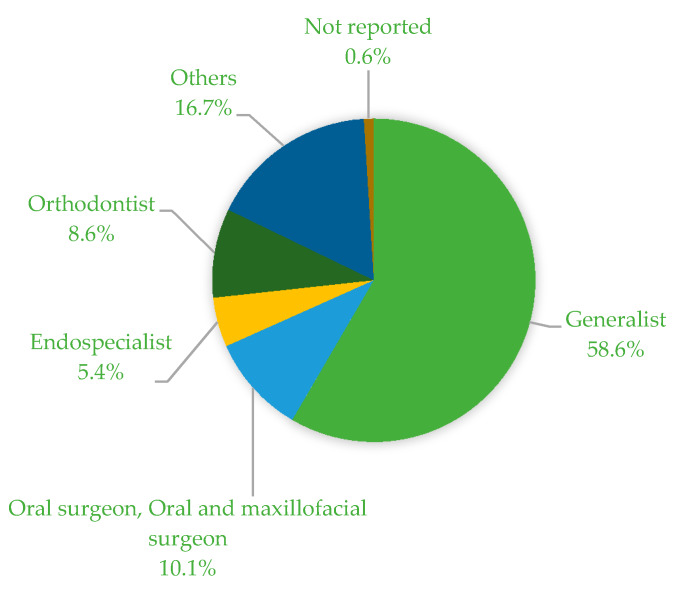
Distribution of the area of their employers’ specialization of the study population (in %).

Figure 1 shows the distribution of the area of their employers’ specialization. Over half of the respondents (58.6%) worked for generalists. Other fields of work included dental hygiene/dental prophylaxis, prosthetics, pedodontology, and periodontology.

### 3.3. Prevalence of MSDs

**Figure 2:**
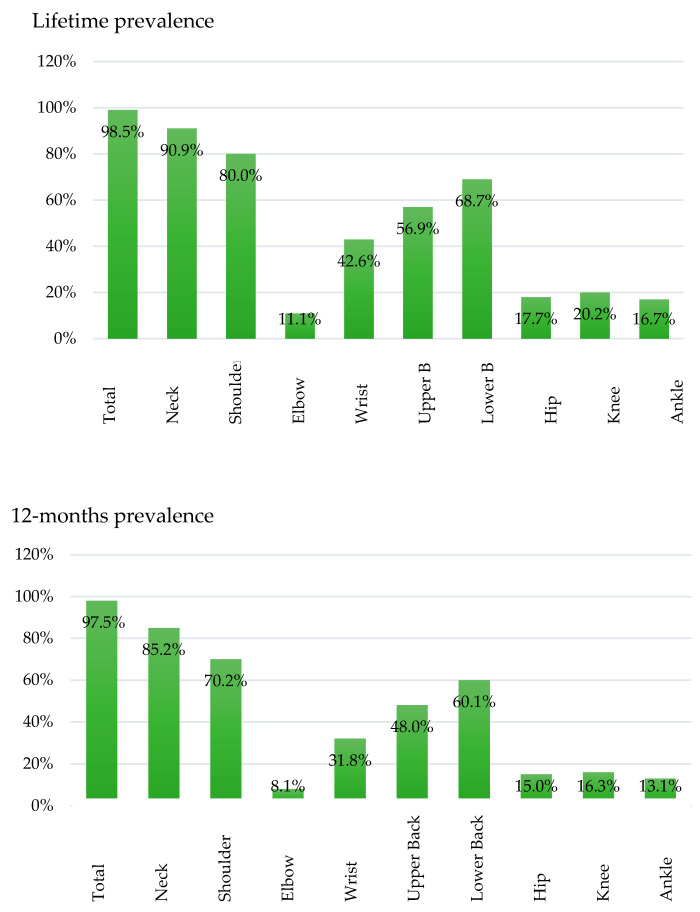

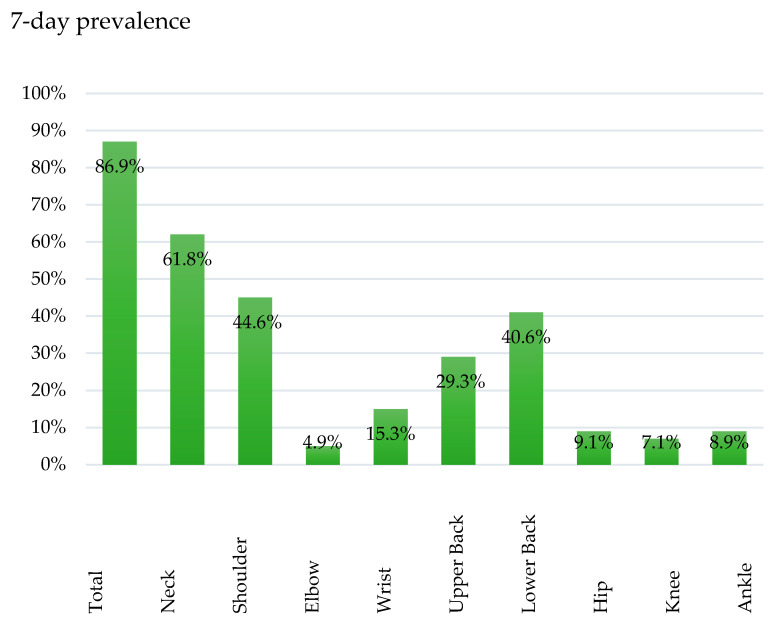
Total prevalence of Musculoskeletal disorder (MSDs) shown in lifetime, 12-month, and seven-day prevalence (in %).

Figure 2 lists the total prevalence of MSDs per region (neck, shoulder, elbow, wrist, upper back, lower back, hip, knee. and ankle) in the order in which they are listed in the Nordic Questionnaire. The prevalence was investigated for the lifetime, 12-months, and seven-days. A total of 98.5% of respondents described at least one MSD-associated complaint at some point in their lives, 97.5% of participants described at least one complaint during the last 12 months, and 86.9% reported at least one complaint in the last seven days. 

The neck was the region that was the most frequently affected body area. Here, 90.9% of individuals reported complaints at some point in their lives, 85.2% in the last 12 months, and 61.8% in the last seven days (Figure 2). The second most frequent region for MDS symptoms was the shoulder. Of the study sample, 80.0% reported shoulder complaints during lifetime, 70.2% in the last 12 months, and 44.6% only in the last 7 days. Besides neck and shoulders, frequent symptoms were also located in the lower back. Of the respondents, 68.7% confirmed a lifetime prevalence, whereas 60.1% noticed lower back symptoms in the last 12 months and 40.6% in the past 7 days. Similarly, complaints of the upper back were often reported. Here, 56.9% stated complaints at some point in their lives, 48.0% in the last 12 months, and 29.3% in the last seven days. Concerning prevalence in the wrists, 42.6% affirmed the question on lifetime, 31.8% on 12-month, and 15.3% on seven-day prevalence. Lifetime complaints in the knees, hips, and ankles were observed by 20.2%, 17.7%, and 16.7% of respondents, respectively. 

Table 3 shows the lifetime, 12-month, and 7-day prevalence of MSDs in the shoulder, elbow, wrist, hip, knee, and ankle, investigated for the left, right, and both sides of the body. For all regions and time periods, the pain prevalence was higher on the right than on the left body side. The only exception was the ankle area. Here, more study participants noticed pain on the left side during the three time periods studied. The prevalence of MSD symptoms in the shoulders was higher on both sides than only on the right or left. During their lifetime, pain in both shoulders was present in 52.0% of individuals (versus in the right side in 20.2% and 7.9% in the left side). The same distribution was seen for the last 12 months (in 43.3% pain in both sides versus 21.2% on the right and 8.1% on the left) or the last 7 days (in 26.1% pain in both sides versus 13.5% on the right and 4.9% on the left). Also, the same pattern was seen for the hip, knee, and ankle regions. For the elbow and the wrist areas, all three prevalences surveyed were the highest on the right side compared to both and the left sides. 

### 3.4. Prevalence of Specific Medical Conditions

Table 4 lists the prevalence of existing medical conditions in our study sample. A total of 34.5% (n = 140) of the subjects reported suffering from one or more of the listed existing medical conditions. Of the questionnaire participants, 16.5% reported a cervical spine syndrome. Of these, 11.3% noticed the beginning of symptoms at an average of 10 years after starting their career. The rates of lumbar spine syndrome, disc prolapse and tendovaginitis were similar across our sample. Of individuals, 13.3% (n = 54) reported a lumbar spine syndrome, 12.6% a disc prolapse (n = 51), and 11.3% a tendovaginitis (n = 46). Furthermore, the rates for osteoarthritis (n = 26, 6.4%) and thoracic spine syndrome (n = 4, 5.9%) were almost the same but lower than the other three conditions. In addition, prevalence rates in the categories rheumatism, carpal tunnel syndrome, flexor tendovaginitis, and cubital tunnel syndrome were in descending order. For all specific medical conditions (except cubital tunnel syndrome), our sampled DAs noticed the beginning of associated complaints more frequently after starting their work as a dental professional than in the time before. 

### 3.5. Correlations between Age/BMI/Height and MSDs 

There was a strong positive correlation between age and work experience (*p* = 0.001, r = 0.92). Due to this highly significant correlation, the correlation between MSDs and work experience can be considered as similar as the correlation between MSDs and age. Therefore, the following correlations were calculated only between the age and the variable of interest. The term “Specific medical conditions” sums up the number of study participants who suffer from at least one or more of the specific medical conditions (Table 5). 

Table 5 presents correlations between specific medical conditions, lifetime prevalence, 12-month, and seven-day prevalence and age, BMI, body weight, and height. The correlation between specific medical conditions and age showed a moderate positive correlation (*p* = 0.001, r = 0.48). We observed a weak, positive correlation between lifetime prevalence of MSDs and age (*p* = 0.04, r = 0.10) and lifetime prevalence of MSDs and body weight (*p* = 0.09, r = 0.09). The 12-month prevalence of MSDs correlated significantly with body height (*p* = 0.04, r = 0.10) and body weight (*p* = 0.02, r = 0.12). The seven-day prevalence of MSD showed a significant positive (weak) correlation with height (*p* = 0.04, r = 0.12). All other correlations were not significant.

For the lifetime and specific medical conditions, the correlation with the BMI, body weight, and body height with the interference factor age (partial correlation) showed no significant results (Table 5).

## 4. Discussion

The aim of the survey was to investigate the prevalence of MSDs in German DAs. The lifetime prevalence of MSDs in this group of dental healthcare professionals was very high with 98.5% of subjects reporting symptoms, 97.5% during the last 12 months, and 86.9% in the last seven days (Table 6). Specifically, the neck, the shoulders, and the lower and upper back were the most affected regions (90%, 80.0%, 68.7%, and 56.9% of respective respondents reported lifetime symptoms). 

The data showed that the prevalence of MSDs in German DAs was very high not only for their lifetime but also during the past 12 months and seven days. MSDs occurred more frequently on the right body side than the left (Table 3). This may be due to the fact that 92.9% of respondents stated to be right-handed (Table 1). The MSDs of the different regions might predispose the DAs for specific medical conditions (Table 4). Almost all of the reported diseases occurred after starting the respondent’s professional life. The most frequent medical conditions were, in descending order, the cervical spine syndrome, lumbar spine syndrome, disc prolapse, and tendovaginitis.

When our results were compared to other studies [16,21,25] our data showed higher rates of MSDs for all investigated body regions. In our sample, neck and back complaints were reported by up to 50.0% and 35.0% [16,21,25]. In addition, the shoulder was up to 43.0% higher than in other studies [16,21,25]. 

The high MSD lifetime prevalence in our study was positively correlated to the age. However, BMI, body weight, and height (excluding the interference factor “age”) did not correlate with the prevalence of MSD in both younger and older participants.

In contrast, only weak correlations could be found between body height and weight (and not for BMI and age) and MSDs for 12-month and seven-day prevalence, so we deduced that an increase, especially in body height, might predict the presence of MSDs. As other studies on DAs did not examine these correlations (BMI, height, weight, age, and MSDs), a direct comparison with other published data was difficult and, therefore, further analysis is necessary to identify other factors that could be possibly associated with such a high prevalence of MSDs. 

Since DAs work closely with dentists in the same working environment, we compared MSDs prevalences in DAs to the MSDs rates stated by dentists [8,9,15,17,18,20,22,26]. In these investigations, either the modified Nordic questionnaire [8,9,18,22] or a questionnaire of similar content was used [15,17,20,26].

Here, the percentage of individuals reporting MDSs was mostly higher in DAs than in dentists. In this study, the 12-month prevalence for the neck was 2.0–47.0% higher than in dentists [8,9,15,18,22]. For the shoulder region, a similar pattern was seen. 

The 12-month prevalence was between 18.0 and 63.0% higher than in dentists’ studies in [9,15,18,26]. Only one Danish study remained an exception since its participants reported an 18% higher 12-month MSD prevalence than was found in this presented DAs’ study [22]. In the lower back, the 12-month prevalence of DAs was similar, with a maximum up to 8.0% higher than the results of the dentists [17,20,22]. In comparison with Czech dentists [18], the available results were about 15.0% lower. The compared MSDs’ 12-month prevalences in the upper back were from similar to 37.0% higher in the current study [15,18].

One assumption why the MSDs’ prevalence is often higher in DAs compared to Ds is that significantly more women work in the profession of DAs. In 2019, 13,671 (97.2%) female DAs compared to 393 (2.8%) male DAs finished their apprenticeship in Germany [52]). Hence, our study sample included almost exclusively women (98.9%). The high prevalence of self-perceived MSDs in DAs might be linked to the high proportion of women in this occupation, as it was shown that women have a different pain perception than men [53]. Generally, individuals have different perceptions of pain, for example, for emotional, situational, or personal reasons [54]. There are also other inter-individual differences with regard to gender [55], height, weight, physical activity, musculature, or any previous illnesses [23]. Further, gender variations in pain perception exist on many levels. Females are more likely to experience a variety of recurrent pains [56] as well as pain of higher severity, frequency, and longer duration compared with men. The higher propensity of the female sex to report pain could reinforce this tendency [57]. However, since the profession of a dental assistant is predominantly performed by women, a realistic gender comparison as well as the comparison between DAs and Ds must be evaluated cautiously. In studies where the prevalence of MSDs was evaluated in male and female dentists, the women reported a higher percentage of MSDs than the male counterparts. Their rates were nevertheless lower than the MSD prevalence in our DA sample. Hence, it is difficult to explain this observation with a greater awareness of females to pain. In view of this fact, the conclusion that DAs suffer more from MSDs than Ds should be taken with caution. 

Conversely, this means that the demonstrably higher sensitivity to pain or greater pain awareness of women [53] could explain why the prevalence was lower in comparative studies with dentists, because both sexes have been analyzed there [9,15,17,18,20,22,26]. In view of this fact, the conclusion that DAs suffer more from MSDs than Ds should be taken with caution.

Another fact which might explain the high MSD prevalences among DAs is their working environment. Here, DAs need to adapt to workplace requirements, e.g., in terms of the height at which the patient is placed during treatment or layout of the instruments. As a result, they may be forced to work involuntarily in postures that are less ergonomic and consequently more prone to result in MSDs. In this context, the difference in body height between a male dentist and a female dentist assistant should be considered. Basically, DAs probably need to be more attuned to the dentists’ working posture than the other way around. Furthermore, the percentage of the time working at a patient in relation to daily work hours might be higher in DAs compared with Ds, who are responsible for more administrative tasks than DAs (Table 2).

This survey showed a high prevalence of MSDs among almost exclusively female dental professionals. In comparison to studies with dentists of both sexes, high percentages of MSDs in dental healthcare professionals prevail—independent of the gender, but less than in DAs. However, in order to better understand the study results, the data were compared to the MSD prevalence in the general German population. In two studies, a random sample was inquired by telephone [58,59]. Although no information on the occupation was queried, it can be assumed that a large spectrum of different occupational groups was included. Twenty-five percent of German women and 16.9% of German men reported back pain in the last 12 months, which is a 43.0% and 20.0% lower MSD prevalence compared with the present study. Further, 48.0% of employees noticed neck and back complaints during the last year [59]. In comparison with these results, the 12-month prevalence of MSDs in the neck of DAs was 37.0% higher, in their lower back around 12.0% higher, and in the upper back similarly high, in most categories. It was concluded that the pattern of affected body areas is very similar between the German population and the DA sample population examined in our study. Pain in the lower back, followed by the neck, the shoulder, and the knees [60] is most frequently reported. The absolute prevalence of pain in these regions is higher in the DAs, which might result from the “unhealthy work environment” in dental clinics. Lietz et al. [3] investigated the physical and psychological demands on dental healthcare professionals on an everyday basis. The authors identified work with interruptions, an extraordinary stress level with a high patient volume, and amount of administrative work, as well as vibrations while treating the patient, as predisposing factors for MSDs [3]. But the most impactful factor linked to MSDs was the physically demanding working postures of dental healthcare professionals [3]. When the results of this study were compared to the data of Lietz et al. [3], a higher MSD prevalence was described for DAs in all four predominant pain areas and during the three investigated time periods. This discrepancy may be explained by the heterogeneous groups of dental professionals examined by Lietz et al. [3] compared to our very homogenous group of DAs. 

Individuals, who are suffering pain due to workplace demands and who—as females—are very sensitive to pain, are highly motivated to participate in a study assessing this specific topic. This may translate into a volunteer bias. This, and the nature of accidental sampling of individuals easy to access [61], can lead to biased prevalences and limits the external validity of the study. In addition, younger dental professionals might feel more familiar with the medium of an online questionnaire and are more likely to complete the survey. This could be one reason for the young median age (28 years) of the survey. Further, even if the response rate of online surveys is rising over the past years, the paper-based surveys still have a higher participating quote [62,63]. 

At the moment, the professional apprenticeship of German DAs does not include teaching sessions on ergonomics in the work environment. The results of the present study indicate that related topics should be implemented in the educational program. In order to improve awareness, a preventive view could be strengthened and possible medical conditions could be pointed out at a very early stage of the professional life [64,65,66]. The same applies also for those who were already longer in employment. An approach to the topic of ergonomics in the context of the DA work environment is necessary, i.e., by creating awareness as well as theoretical and practical knowledge, in order to decrease the prevalence and related burden of MSDs. The positive impacts of such training on the MSD prevalence should be analyzed in future studies. Also, investigations of muscle activity, strength, movement, and range of motion during occupational activity are necessary in order to understand the effects of work on the body and the relation to MSDs more deeply. Since such studies have already been carried out for dentists [1], their design should be adapted to DAs. Also, future research could assess preventive steps DAs have already implemented in their workplaces and measure the success of these approaches in reducing MSDs.

## 5. Conclusions

In summary, the prevalence of MSDs in DAs is very high. The most affected area is the neck, followed by the shoulder, the lower back, and the upper back. It, therefore, seems necessary to devote more attention to ergonomics in the workplace of DAs as well as to teach this topic along with preventive measures during their apprenticeship. However, the results of this study must be seen critically since mostly women answered this questionnaire. Since it is known that women have a greater pain awareness than men, a comparison with existing studies, which also include male subjects, has significant limitations. 

## Figures and Tables

**Table 1:** Personal characteristics data of the study population. In addition to the number (n) of participants, the interquartile distance (I50) was also shown, if necessary.

Personal Characteristics	DAs *
Sex	
Female	401 (98.8%)
Male	5 (1.2%)
Age (Years)	
$\tilde{x}$ (I50)	28 (15)
Height (cm)	
$\tilde{x}$ (I50)	166 (8)
Not reported in n (%)	4 (1.0%)
Weight (kg)	
$\tilde{x}$ (I50)	66 (18)
Not reported in n (%)	5 (1.2%)
BMI (kg/m^2^)	
$\tilde{x}$ (I50)	23.9 (5.8)
Not reported in n (%)	8 (2.0%)
Handedness	
Right	377 (92.9%)
Left	29 (7.1%)

* DAs: Dental assistants.

**Table 2:** Work -related characteristics of the study population.

Work-Related Parameter	Das *
Professional group	
n (%)	
Qualified dental assistant	322 (79.3)
Trainee qualified dental assistant	84 (20.7)
Professional experience in years	
$\tilde{x}$ (I50)	8 (15)
Not reported in n (%)	19 (4.7)
Total working time by the week in hours	
$\tilde{x}$ (I50)	38.5 (8)
Not reported in n (%)	28 (6.9)
Treatment time by the week in hours	
$\tilde{x}$ (I50)	30 (15)
Not reported in n (%)	35 (8.6)
Administrative time by the week in hours	
$\tilde{x}$ (I50)	4 (7)
Not reported in n (%)	39 (9.6)

* DAs: Dental assistants.

**Table 3:** Right and left MSDs’ prevalence of shoulder, elbow, wrist, hip, knee, and ankle shown in lifetime, 12-month, and seven-day prevalence.

Body Regions		Lifetime Prevalence n (%)	12-Month Prevalence n (%)	7-Day Prevalence n (%)
Shoulder	Left	32 (7.9)	33 (8.1)	20 (4.9)
	Right	82 (20.2)	86 (21.2)	55 (13.5)
	Both	211 (52.0)	176 (43.3)	106 (26.1)
Elbow	Left	8 (2.0)	2 (0.5)	1 (0.2)
	Right	25 (6.2)	20 (4.9)	13 (3.2)
	Both	12 (3.0)	11 (2.7)	6 (1.5)
Wrist	Left	15 (3.7)	13 (3.2)	5 (1.2)
	Right	87 (21.4)	70 (17.2)	29 (7.1)
	Both	71 (17.5)	52 (12.8)	28 (6.9)
Hip	Left	9 (2.2)	10 (2.5)	8 (2)
	Right	22 (5.4)	20 (4.9)	10 (2.5)
	Both	41 (10.1)	33 (8.1)	19 (4.7)
Knee	Left	19 (4.7)	19 (4.7)	9 (2.2)
	Right	20 (4.9)	22 (5.4)	10 (2.5)
	Both	43 (10.6)	29 (7.1)	10 (2.5)
Ankle	Left	11 (2.7)	9 (2.2)	6 (1.5)
	Right	9 (2.2)	5 (1.2)	5 (1.2)
	Both	48 (11.8)	39 (9.6)	25 (6.2)

**Table 4:** Occurrence and start of occurrence of specific medical conditions.

Disease	Occurrence n (%)	Occurrence before Start of Profession n (%)	Occurence after Start of Profession n (%)	Occurence after xx Years $\tilde{x}$
Total	140 (34.5)			
Rheumatism	12 (3.0)	2 (0.5)	7 (1.7)	15 (20)
Osteoarthritis	26 (6.4)	1 (0.2)	20 (4.9)	18 (13)
Carpal Tunnel Syndrome	12 (3.0)		8 (2.0)	7 (16)
Disc Prolapse	51 (12.6)	2 (0.5)	31 (7.6)	12 (15)
Tendovaginitis	46 (11.3)	8 (2.0)	24 (5.9)	4 (7)
Flexor Tendovaginitis	6 (1.5)	1 (0.2)	3 (0.7)	14.5 (19)
Cubital Tunnel Syndrome	1 (0.2)	0	1 (0.2)	0
Cervical Spine Syndrome	67 (16.5)	9 (2.2)	46 (11.3)	10 (10)
Thoracic Spine Syndrome	24 (5.9)	4 (1.0)	14 (3.4)	10 (10)
Lumbar Spine Syndrome	54 (13.3)	3 (0.7)	33 (8.1)	10 (12)

**Table 5:** Correlation between age/Body-Mass-Index (BMI)/body height/body weight and the number of complaint regions in lifetime prevalence, 12-month prevalence, and seven-day prevalence of MSD as well as the number of specific medical conditions. For the lifetime prevalence and specific medical conditions a partial correlation was calculated corrected for the interference factor age. Significant correlations are marked with an asterisk.

Correlation Parameter	Age		BMI		Body Height		Body Weight	
	Spearman Correlation Coefficient r	*p*-Value	Spearman Correlation Coefficient r	*p*-Value	Spearman Correlation Coefficient r	*p*-Value	Spearman Correlation Coefficient r	*p*-Value
Specific medical Conditions (in total)	0.48	0.001 *	−0.03	0.59	0.04	0.37	0.001	0.99
Lifetime prevalence of MSD (in total)	0.10	0.04 *	0.07	0.19	0.06	0.24	0.09	0.09
12-month prevalence of MSD (in total)	0.05	0.37	0.08	0.12	0.10	0.04 *	0.12	0.02 *
7-day prevalence of MSD (in total)	−0.01	0.88	−0.03	0.55	0.12	0.04 *	0.01	0.83

**Table 6:** Most frequent prevalences of MSDs among dental assistants in Germany (in %).

Body Regions	Lifetime Prevalence	12-Month Prevalence	7-Day Prevalence
Total	98.5	97.5	86.9
Neck	90.9	85.2	61.8
Shoulder	80.0	70.2	44.6
Upper back	56.9	48.0	29.3
Lower Back	68.7	60.1	40.6
